# The prognostic value of 18-FDG positron emission tomography in T cell non-Hodgkin lymphoma

**DOI:** 10.1038/bcj.2017.39

**Published:** 2017-04-21

**Authors:** R Eslick, L Dunlop, M Lin, D Hsu, S Ling

**Affiliations:** 1 Department of Haematology, Liverpool Hospital, Sydney, New South Wales, Australia; 2 Department of Nuclear Medicine and PET, Liverpool Hospital, Sydney, New South Wales, Australia

18F-fluorodeoxyglucose positron emission tomography (FDG-PET) scans are typically positive in patients with T cell non-Hodgkin lymphoma (T-NHL) and can assist in the diagnosis and staging of this uncommon malignancy. Previous studies have suggested that FDG-PET is a useful tool in initial staging of T-NHL; however, there is relatively little evidence for the role of the post-treatment FDG-PET in this patient population. Some published studies support the role of a negative post-treatment PET in predicting progression-free survival (PFS) and overall survival (OS),^1, 2^ while other studies have found that a negative interim or post-treatment PET does not predict outcome.^3, 4^ Recently published guidelines remain ambivalent about the utility of the PET scan in T-NHL, stating ‘there is no clearly defined role for FDG-PET in this disease group'.^5^ We aimed to evaluate the predictive value of interim and post-treatment PET scans in T-NHL to determine their effect on PFS and OS.

Patients who underwent a PET scan for a diagnosis of T-NHL from 2002 to May 2016 were retrospectively identified by a search of the medical records and NSW Cancer Registry in a single tertiary hospital. Demographics, treatments and survival outcomes were recorded in a de-identified database. Final PET results were correlated with PFS and OS outcomes. Ethics approval was obtained for the study. Kaplan−Meier survival analysis and the log−rank test were used to assess the difference in survival with a *P-*value of <0.05 to indicate statistical significance.

A total of 47 patients were identified as eligible for inclusion, 29 were male (62%), with an average age of 52 years at diagnosis. Out of the total patients, 45 (96%) had an initial PET, 26 (62%) had an interim PET, and 39 (83%) had a post-treatment PET. The majority of patients had advanced disease at diagnosis, with an Ann Arbor stage of III or IV (70%) and an international prognostic index score of 2 or above (60%). The frequency of specific T cell histologies were anaplastic large cell, ALK-1 positive (15%), ALK-1 negative (24%), and unspecified (2%); peripheral T cell lymphoma not otherwise specified (28%); angioimmunoblastic T cell lymphoma (11%); subcutaneous panniculitis-like T cell lymphoma (4%); mycosis fungoides (4%); and others (6%). The majority of patients were treated with CHOP (cyclophosphamide, doxorubicin, vincristine and prednisone, 66%), with other chemotherapy regimens including CHOP with etoposide, HyperCVAD (cyclophosphamide, doxorubicin, vincristine, dexamethasone, methotrexate and cytarabine), SMILE (dexamethasone, methotrexate, ifosfamide, L-asparaginase and etoposide), and cyclosporine with prednisone. Radiotherapy alone was given in 6% of cases. Patients were followed up for a mean of 33.8 months. During the follow-up period, 22 patients progressed (47%) and 20 died (43%).

Those with a positive post-treatment PET scan (*n*=11) had a median OS of 27.9 months (Figure 1). OS was not reached for those with a negative post-treatment scan (*P*=0.0017). The median PFS for those with a positive post-treatment PET scan was 5.2 months, with PFS not reached for those with a negative scan (*P*=0.0012; Figure 2). The interim PET scan did not appear to be significant in predicting PFS or OS in our cohort.

The post-treatment PET scan appears to be of value in predicting both PFS and OS in T-NHL. Our study is limited by the low patient numbers due to disease rarity and the inherently heterogenous behaviour of the different subtypes of T-NHL. The retrospective, single-centre nature of the study is also an inherent limitation. Nevertheless, this study adds to the growing body of evidence supporting the importance of the post-treatment PET scan in predicting outcomes in T-NHL.

## Figures and Tables

**Figure 1:**
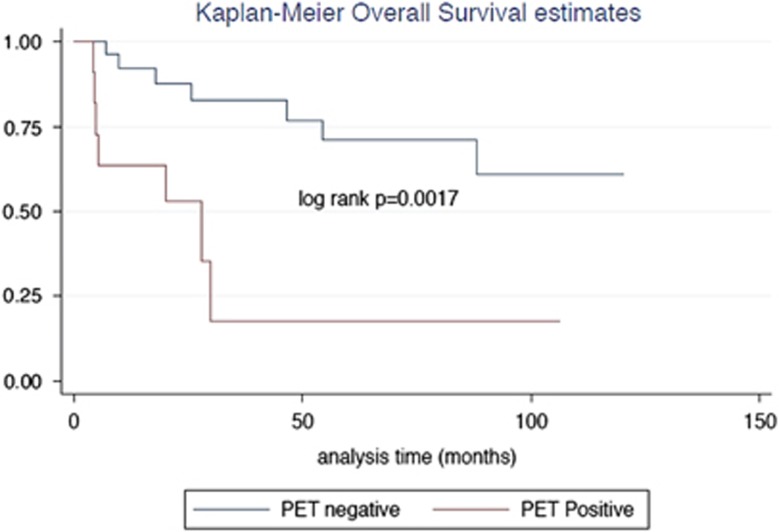
Kaplan−Meier overall survival curves for those with a negative post-treatment PET, compared to those with a positive post-treatment PET.

**Figure 2:**
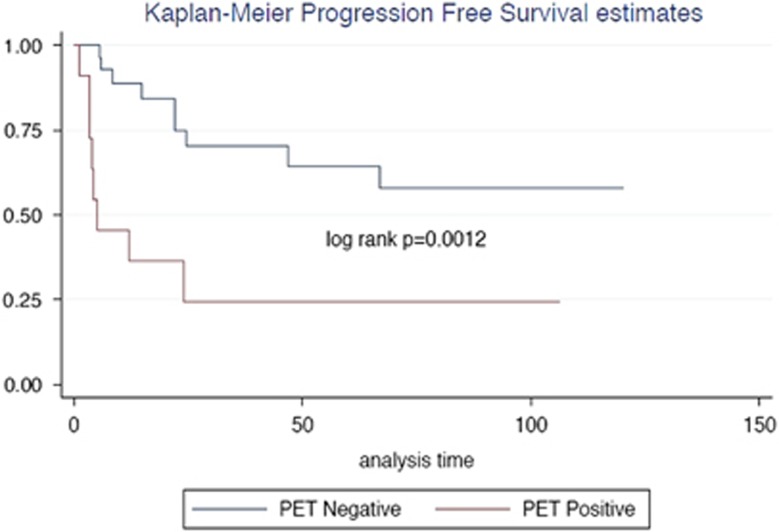
Progression-free Kaplan−Meier survival curves for those with a negative post-treatment PET, compared to those with a positive post-treatment PET.
